# Retraction: Impact of some pyrrolidinium ionic liquids on copper dissolution behavior in acidic environment: experimental, morphological and theoretical insights

**DOI:** 10.1039/d5ra90075a

**Published:** 2025-06-11

**Authors:** Emad E. El-Katori, Ashraf S. Abousalem

**Affiliations:** a Department of Chemistry, Faculty of Science, The New Valley University El-Kharja-72511 Egypt emad_992002@yahoo.com emad.elkatori@scinv.au.edu.eg +20-927925393 +20-1023318210; b Chemistry Department, Faculty of Science, Mansoura University Egypt

## Abstract

Retraction of ‘Impact of some pyrrolidinium ionic liquids on copper dissolution behavior in acidic environment: experimental, morphological and theoretical insights’ by Emad E. El-Katori *et al.*, *RSC Adv.*, 2019, **9**, 20760–20777, DOI: https://doi.org/10.1039/C9RA03603B.

The Royal Society of Chemistry hereby wholly retracts this *RSC Advances* article due to concerns with the reliability of the data.

The AFM images in Fig. 10A and B were the same as those published by the authors in ref. 1. The AFM images in Fig. 10C and D were the same as those previously published by a different author in ref. 2. The authors stated that the author of ref. 2 was part of their research group and the AFM imaging was carried out outside the faculty and was distributed across the research group.

The authors alerted us to this error and requested a correction for these images, however given the significance of these concerns, the Editor has lost confidence that the findings presented in this paper are reliable.

The authors regret that the affiliation of the 2nd author was incorrect in the original version, and the affiliation where the work was conducted in shown above.

This retraction supersedes the information provided in the Expression of Concern related to this article.

The authors were informed about the retraction of the article, Emad E. El-Katori disagreed with the retraction on behalf of all authors, but the other author has not indicated whether they agree with the decision to retract.

Signed: Laura Fisher, Executive Editor, *RSC Advances*

Date: 4th June 2025

**References**

1 M. M. Abdou, O. Younis and E. E. El-Katori, *J. Mol. Liq.*, 2022, 119506, DOI: 10.1016/j.molliq.2022.119506.

2 A. S. Fouda, *Int. J. Electrochem. Sci.*, 2018, 4670–4692, DOI: 10.20964/2018.05.28.

